# A Chapter of Heredity

**Published:** 1905-08-26

**Authors:** 


### A CHAPTER OF HEREDITY.

The patience and enterprise of M. V. Galippe[1] has provided him with material for an interesting and out-of-the-way contribution to the curious workings of heredity. He states that "teratological heredity," by which he means morphological characters amounting almost to an anomaly, are subject to the same laws that govern the pathological inheritances which are more generally in the eye of our profession. He observes that even in the ordinary course of life one may see peculiarities of feature, attitude, or form, handed down from generation to generation. In the animal world it is a commonplace that "teratological families" may be produced by selection, a fact of which the monstrous physical characters of the bulldog form a standing and conspicuous example.

It has occurred to the author that the families most likely to afford good evidence of this perpetuation of physical peculiarities are those of royal houses whose ancestry is well known, and whose features are comprehensively recorded in stone or upon canvas. Such a line will provide a particularly stringent test, since it is well known that royal unions are determined by natural selection to a degree far less than are those of common folk the claims of policy forming so prominent a feature in the contracting of royal marriages.

After a careful consideration of available data M. Galippe has established a teratological peculiarity in the House of Hapsburg. It consists of a well-marked prognathism, associated with an exaggerated prominence of the lower lip. This peculiarity is an excellent one for the author's purpose, since it is easy of recognition in statue or picture, and a prolonged study has assured the author that its presence is almost constant in the Hapsburgs. He observes that all representations, especially of kings and royal persons, must be regarded with a severe scepticism, in view of the tendency of art to embellish nature by the mitigation of unprepossessing elements of feature or form. He considers that sculptors and medallists are less prone to flattery than are painters, and that the sin of painters is further exaggerated by those who engrave from their portraits. It is not surprising to hear that this humane but unscientific tendency must be discounted to a still larger extent when the model is a lady.

With a due recognition of the necessary qualifications imposed by these artistic delinquencies the author considers himself able to trace the characteristic feature of the Hapsburgs almost without intermission from the fifteenth century onwards. It appears to intermit occasionally, but inevitably reappears, especially in males. None the less, the women of the house, who themselves possess the peculiarity to a modified extent only, appear to have been able to impose it with unabated prominence upon their descendants, no matter whom they happen to have married. But it is in the case of consanguine marriages within the house itself that the most striking examples of this heredity are manifested; which, of course, is what one would expect.

The application to history of the facts above stated opens up some interesting lines of illustration, and some of speculation not untinged with scandal. Napoleon I., having divorced Joséphine on account of her childlessness, married an archduchess of the Hapsburg house to help him found his family. His first child by the marriage was no Napoleon, but an undoubted Hapsburg in the formation of his skull. This son is he who was known as the King of Rome. Again, the Emperor Charles V. of Germany is well known for the features he imposed upon his descendants. These were transmitted without interruption, with one striking exception. Don Juan of Austria, who was by common acceptation an illegitimate son of the Emperor, was anything but a Hapsburg, and entirely unlike his brother Philip II. The prognathous face was lacking in Don Juan, and M. Galippe is strongly of opinion that the great Emperor was deceived in imagining the bastard to be his own issue. This is particularly likely to be a just criticism, since the mother of Don Juan showed herself at a later time to be a woman of such infamous morals that her son was led to have her imprisoned in a convent.

Another interesting application of the doctrine detailed above is made in the matter of the descendants of Louis XVI. Upon the murder of the latter, his second son Charles, Louis XVII., continued in prison till his death, which was followed by the appearance of many false dauphins. One of these was a Potsdam watchmaker, by name Naundorff, whose claim rested on a striking resemblance to the Bourbon features. Now both Louis XVI. and Marie Antoinette were Hapsburgs, and both showed the family prognathism; it might have been expected that their descendants would have been on this account doubly endowed, and M. Galippe considers that Louis XVII. was undoubtedly so favoured, though not to any remarkable degree. Naundorff, on the other hand, exhibited no trace of prognathism, and our author's investigations have revealed none in his descendants, facts which add assurance to the falsity of his pretensions.

[1] Bull. de l'Acad. de Méd. July 4, 1905.

